# Evolution and Complexity of Micro RNA in the Human Brain

**DOI:** 10.3389/fgene.2012.00166

**Published:** 2012-09-03

**Authors:** Walter J. Lukiw

**Affiliations:** ^1^Neurology, Neuroscience and Ophthalmology, Louisiana State University Health Sciences CenterNew Orleans, LA, USA

The discovery of small non-coding RNAs (sncRNAs), and the biological actions of these ribonucleotide entities in the developing, aging, and pathological human central nervous system (CNS) has opened a novel and fascinating vista into our appreciation of human brain epigenetics, the role of sncRNAs on homeostatic and pathogenic gene control, and the potential role of single stranded, 22&nt signals in shaping the transcriptome of the human CNS (Lukiw et al., 1992; Ambros, 2004, 2011; Lukiw, 2007; Lukiw and Pogue, 2007; Guo et al., 2010). Indeed, a major family of sncRNAs known as micro RNAs (miRNAs) has emerged as essential and critical regulators of gene expression in diverse neurobiological pathways in both health and disease. The primary mode of action of miRNAs is to bind to target ribonucleotide sequences in messenger RNAs (mRNAs), typically in their 3′ un-translated region (3′-UTR), resulting in a highly selective repression of the readout of that mRNA's genetic information (Lukiw, 2007; Guo et al., 2010; Ambros, 2011). Pathogenically up-regulated miRNAs can be considered an epigenetic mechanism to down-regulate specific mRNAs and their expression. Up-regulated miRNA in human brain-specific neurodegenerative disorders such as Alzheimer's disease (AD) may help explain the large number of essential brain gene messages observed to be significantly down-regulated in AD affected anatomical regions, such as in the temporal lobe neocortex (Brodmann area 22) and hippocampus, or in primary human brain cell cultures that are chronically subjected to AD-relevant stressors (Loring et al., 2001; Colangelo et al., 2002; Lukiw, 2004, 2012; Lukiw and Pogue, 2007; Sethi and Lukiw, 2009; Ginsberg et al., 2012; Lukiw and Alexandrov, 2012; Lukiw et al., 2012; Wang et al., 2012). While the total number of human miRNAs identified to date in all tissues numbers around 2000, the number of abundant human brain miRNAs is surprisingly far less; for example only about one-thirtieth of all so far identified human miRNAs are abundantly present in the human brain association neocortex (Table 1). Further, mathematical, bioinformatical, and nucleotide sequence analysis indicate that a 22&nt single stranded RNA composed of four different ribonucleotides (A, G, C, and U) may have as many as 10^13^ possible sequence combinations, so the fact that there typically only on the scale of ∼10^2^ abundant miRNAs in any single human brain sample suggests a very high developmental and evolutionary selection pressure to utilize only specific miRNA oligonucleotide sequences that will yield neurobiologically useful miRNA–mRNA interactions (Lukiw, 2012; Lukiw and Alexandrov, 2012). Table 1 lists the 61 most abundant miRNAs found in a critically controlled pool of short post-mortem interval (PMI; <3&h; total *N*&=&21) human association neocortex (Brodmann area A22) *indicating that only about 61 of 10^13^, or less than one in one hundred trillion of all potential miRNA species possible have been selected to play some role in brain-essential regulatory functions as a brain abundant miRNA*. An additional interesting fact is that miRNAs are highly developmental stage-, tissue- and cell-specific, even in adjacent cell types, and mean abundance of these individual species may fluctuate as do the levels of transcription factors which regulate pre-miRNA transcription (Burmistrova et al., 2007; Lukiw and Pogue, 2007; Guo et al., 2010; Lukiw and Alexandrov, 2012). The stability, and hence persistence of action, of sncRNAs, and miRNA appear to follow the adenine-uracil (AU) element (ARE) rich rules as for mRNA stability, and the half-life of several brain abundant miRNAs has been observed to be surprisingly restricted (Sethi and Lukiw, 2009; Wang et al., 2012). For example, human brain miRNA half-lives have been estimated to range from about 30&min (miRNA-9) to about 3&h (miRNA-125b), so overall the generation of a new miRNA, its longevity and interdiction of translational processes may be relatively rapid neurogenetic process (Sethi and Lukiw, 2009). However, other biophysical and neurogenetic parameters such as miRNA secondary and tertiary structure, miRNA binding proteins, and other factors may significantly extend miRNA viability and action.

**Table 1:** **Most abundant miRNAs in the human brain temporal lobe association neocortex; ranked by miRNA numerical designation; based on pooled data from *N* = 21 short post-mortem interval (PMI) controls; this group included 9 males and 12 females, mean age 72 ± 6.3 years; all PMIs less than 3 h; all of these miRNAs yielded a relative signal strength of 5000 or greater on an LC Sciences miRNA array Genechip (proprietary MRA-1001-miRNA microfluidic chip analytical platform; 1922 small RNAs analyzed; LC Sciences Corporation, Houston, TX, USA) and ranked the top 61 of most abundant miRNAs detected; except for miRNAs listed here, all other miRNAs are in a significantly lower abundance; note high basal abundance of the let-7a to let-7g group of miRNAs; note that AD-abundant miRNAs such as miRNA-155 do not appear in this table as in control brain they are relatively scarce, but are significantly induced as in AD brain, and especially as AD progresses**.

hsa-let-7a-5p	hsa-miR-23b-3p
hsa-let-7b-5p	hsa-miR-23c
hsa-let-7c	hsa-miR-24-3p
hsa-let-7d-5p	hsa-miR-26a-5p
hsa-let-7f-5p	hsa-miR-26b-5p
hsa-let-7g-5p	hsa-miR-27a-3p
hsa-miR-100-5p	hsa-miR-27b-3p
hsa-miR-101-3p	hsa-miR-29a-3p
hsa-miR-103a-3p	hsa-miR-29b-3p
hsa-miR-107	hsa-miR-29c-3p
hsa-miR-124-3p	hsa-miR-30a-5p
hsa-miR-125a-5p	hsa-miR-30b-5p
hsa-miR-125b-5p	hsa-miR-30c-5p
hsa-miR-126-3p	hsa-miR-30d-5p
hsa-miR-1273g-3p	hsa-miR-338-3p
hsa-miR-128	hsa-miR-342-3p
hsa-miR-139-5p	hsa-miR-3665
hsa-miR-145-5p	hsa-miR-3960
has-miR-146a-5p	hsa-miR-4324
hsa-miR-151a-5p	hsa-miR-4454
hsa-miR-151b	hsa-miR-4516
hsa-miR-16-5p	hsa-miR-451a
hsa-miR-181a-5p	hsa-miR-4787-5p
hsa-miR-181b-5p	hsa-miR-5001-5p
hsa-miR-181d	hsa-miR-5100
hsa-miR-191-5p	hsa-miR-574-3p
hsa-miR-195-5p	hsa-miR-9-3p
hsa-miR-21-5p	hsa-miR-9-5p
hsa-miR-221-3p	hsa-miR-99a-5p
hsa-miR-222-3p	hsa-miR-99b-5p
hsa-miR-23a-3p	

*Note that miRNAs are developmentally and temporally regulated and may display variable abundance in different cells or cell types; larger sample sets need to be analyzed to research these abundance patterns; at this point we cannot exclude the abundance and complexity of other enriched miRNA species. See also Burmistrova et al. (2007)*.

Another poorly understood layer of complexity in miRNA signaling is that several miRNAs may regulate a single mRNA in a neurogenetic control process known as miRNA *convergence*, and conversely, a single miRNA may interact with multiple mRNAs in a regulatory process known as miRNA *divergence* (Lukiw and Alexandrov, 2012). Multiple functioning, and sometimes overlapping, miRNA binding sites have been found, for example, in the 232&nt 3′-UTR of the human innate immune response regulator complement factor H (CFH) mRNA in the human brain and retina. Therefore a different repertoire of miRNAs might be used to regulate CFH expression in either of these related tissue types (Lukiw and Alexandrov, 2012; Lukiw et al., 2012). Indeed, overlapping of miRNA binding sites in the 3′-UTR of the same mRNA may be an evolutionary strategy to regulate specific gene expression via a common genetic signal in multiple and related cell types (Lukiw and Alexandrov, 2012; Lukiw et al., 2012). Anti-miRNA (antagomir, AM) approaches to these multiple miRNA binding sites in mRNA 3′-UTRs are currently redirecting our therapeutic perspectives to potentially yield novel and highly efficacious treatment strategies for brain diseases that have not yet been considered (Lukiw et al., 2008, 2012; Cui et al., 2010; Wang et al., 2012). It will be particularly interesting to analyze global miRNA–mRNA targeting and control patterns in the brain and CNS to further unravel this fascinating, evolutionarily selective regulatory networking. How individual sncRNA, miRNA, and mRNA complexity together become progressively altered in aging and disease, and their selective quenching using novel AM strategies should be of high therapeutic value in the homeostatic stabilization of miRNA complexity, and in the treatment of common, age-related degenerative disorders, both inside and outside of the central nervous system.

## References

[B1] Ambros V. (2004). The functions of animal microRNAs. Nature 431, 350–355 10.1038/nature02871 15372042

[B2] Ambros V. (2011). MicroRNAs and developmental timing. Curr. Opin. Genet. Dev. 21, 511–517 10.1016/j.gde.2011.04.003 21530229 PMC3149784

[B3] Burmistrova O. A. Goltsov A. Y. Abramova L. I. Kaleda V. G. Orlova V. A. Rogaev E. I. (2007). MicroRNA in schizophrenia: genetic and expression analysis of miR-130b (22q11). Biochemistry Mosc. 72, 578–582 10.1134/S0006297907050161 17573714

[B4] Colangelo V. Schurr J. Ball M. J. Pelaez R. P. Lukiw W. J. (2002). Gene expression profiling of 12633 genes in Alzheimer hippocampal CA1: transcription and neurotrophic factor down-regulation and up-regulation of apoptotic and pro-inflammatory signaling. J. Neurosci. Res. 70, 462–473 10.1002/jnr.10351 12391607

[B5] Cui J. G. Li Y. Y. Zhao Y. Bhattacharjee S. Lukiw W. J. (2010). Differential regulation of interleukin-1 receptor-associated kinase-1 (IRAK-1) and IRAK-2 by miRNA-146a and NF-kB in stressed human astroglial cells and in Alzheimer disease. J. Biol. Chem. 285, 38951–38960 10.1074/jbc.M110.147819 20937840 PMC2998119

[B6] Ginsberg S. D. Alldred M. J. Che S. (2012). Gene expression levels assessed by CA1 pyramidal neuron and regional hippocampal dissections in Alzheimer's disease. Neurobiol. Dis. 45, 99–107 21821124 10.1016/j.nbd.2011.07.013 PMC3220746

[B7] Guo H. Ingolia N. T. Weissman J. S. Bartel D. P. (2010). Mammalian microRNAs predominantly act to decrease target mRNA levels. Nature 466, 835–840 10.1038/nature09267 20703300 PMC2990499

[B8] Loring J. F. Wen X. Lee J. M. Seilhamer J. Somogyi R. (2001). A gene expression profile of Alzheimer's disease. DNA Cell Biol. 20, 683–695 10.1089/10445490152717541 11788046

[B9] Lukiw W. J. (2004). Gene expression profiling in fetal, aged, and Alzheimer hippocampus: a continuum of stress-related signaling. Neurochem. Res. 29, 1287–1297 10.1023/B:NERE.0000023615.89699.63 15176485

[B10] Lukiw W. J. (2007). Micro-RNA speciation in fetal, adult and Alzheimer's disease hippocampus. Neuroreport 18, 297–300 10.1097/WNR.0b013e3280148e8b 17314675

[B11] Lukiw W. J. (2012). NF-KB-regulated micro RNAs (miRNAs) in primary human brain cells. Exp. Neurol. 235, 484–490 10.1016/j.expneurol.2011.11.022 22138609 PMC3321120

[B12] Lukiw W. J. Alexandrov P. N. (2012). Regulation of complement factor H (CFH) by Multiple miRNAs in Alzheimer's disease (AD) brain. Mol. Neurobiol. [Epub ahead of print]. 22302353 10.1007/s12035-012-8234-4 PMC3703615

[B13] Lukiw W. J. Handley P. Wong L. McLachlan D. R. C. (1992). BC200 and other small RNAs in normal human neocortex, non-Alzheimer dementia (NAD), and senile dementia of the Alzheimer type (AD). Neurochem. Res. 17, 591–597 10.1007/BF00968788 1603265

[B14] Lukiw W. J. Pogue A. I. (2007). Induction of specific miRNA species by ROS-generating metal sulfates in primary human brain cells. J. Inorg. Biochem. 101, 1265–1269 10.1016/j.jinorgbio.2007.06.004 17629564 PMC2080079

[B15] Lukiw W. J. Surjyadipta B. Dua P. Alexandrov P. N. (2012). Common micro RNAs (miRNAs) target complement factor H (CFH) regulation in Alzheimer's disease (AD) and in age-related macular degeneration (AMD). Int. J. Biochem. Mol. Biol. 3, 105–116 22509485 PMC3325769

[B16] Lukiw W. J. Zhao Y. Cui J. G. (2008). An NF-kB-sensitive micro RNA-146a-mediated inflammatory circuit in Alzheimer disease and in stressed human neural cells. J. Biol. Chem. 283, 31315–31322 10.1074/jbc.M805371200 18801740 PMC2581572

[B17] Sethi P. Lukiw W. J. (2009). Micro-RNA (miRNA) abundance and stability in human primary brain cells and in human brain tissues. Neurosci. Lett. 459, 100–104 10.1016/j.neulet.2009.04.052 19406203

[B18] Wang H. Chiu M. Xie Z. Chiu M. Liu Z. Chen P. Liu S. Byrd J. C. Muthusamy N. Garzon R. Croce C. M. Marcucci G. Chan K. K. (2012). Synthetic microRNA cassette dosing: pharmacokinetics, tissue distribution and bioactivity. Mol. Pharm. 9, 1638–1644 10.1021/mp300023x 22574727 PMC3977775

